# Comparative transcriptome analysis reveals the potential mechanism of GA_3_-induced dormancy release in *Suaeda glauca* black seeds

**DOI:** 10.3389/fpls.2024.1354141

**Published:** 2024-06-11

**Authors:** Hongfei Wang, Tianjiao Xu, Yongjia Li, Rui Gao, Xuelin Tao, Jieqiong Song, Changping Li, Qiuli Li

**Affiliations:** ^1^ The Key Laboratory of Plant Biotechnology of Liaoning Province, School of Life Science, Liaoning Normal University, Dalian, China; ^2^ Dandong Forestry and Grassland Development Service Center, Dandong, China

**Keywords:** *Suaeda glauca*, exogenous gibberellin, dormancy release, transcriptome, differentially expressed genes

## Abstract

*Suaeda glauca* Bunge produces dimorphic seeds on the same plant, with brown seeds displaying non-dormant characteristics and black seeds exhibiting intermediate physiological dormancy traits. Previous studies have shown that black seeds have a very low germination rate under natural conditions, but exogenous GA_3_ effectively enhanced the germination rate of black seeds. However, the physiological and molecular mechanisms underlying the effects of GA_3_ on *S. glauca* black seeds are still unclear. In this study, transcriptomic profiles of seeds at different germination stages with and without GA_3_ treatment were analyzed and compared, and the TTF, H_2_O_2_, O_2_^–^, starch, and soluble sugar contents of the corresponding seed samples were determined. The results indicated that exogenous GA_3_ treatment significantly increased seed vigor, H_2_O_2_, and O_2_^–^ contents but decreased starch and soluble sugar contents of *S. glauca* black seeds during seed dormancy release. RNA-seq results showed that a total of 1136 DEGs were identified in three comparison groups and were involved mainly in plant hormone signal transduction, diterpenoid biosynthesis, flavonoid biosynthesis, phenylpropanoid biosynthesis, and carbohydrate metabolism pathway. Among them, the DEGs related to diterpenoid biosynthesis (*SgGA3ox1*, *SgKAO* and *SgGA2ox8*) and ABA signal transduction (*SgPP2Cs*) could play important roles during seed dormancy release. Most genes involved in phenylpropanoid biosynthesis were activated under GA_3_ treatment conditions, especially many *SgPER* genes encoding peroxidase. In addition, exogenous GA_3_ treatment also significantly enhanced the expression of genes involved in flavonoid synthesis, which might be beneficial to seed dormancy release. In accordance with the decline in starch and soluble sugar contents, 15 genes involved in carbohydrate metabolism were significantly up-regulated during GA_3_-induced dormancy release, such as *SgBAM*, *SgHXK2*, and *SgAGLU*, etc. In a word, exogenous GA_3_ effectively increased the germination rate and seed vigor of *S. glauca* black seeds by mediating the metabolic process or signal transduction of plant hormones, phenylpropanoid and flavonoid biosynthesis, and carbohydrate metabolism processes. Our results provide novel insights into the transcriptional regulation mechanism of exogenous GA_3_ on the dormancy release of *S. glauca* black seeds. The candidate genes identified in this study may be further studied and used to enrich our knowledge of seed dormancy and germination.

## Introduction

Seed dormancy is usually defined as the failure of an intact, viable seed to germinate under favorable environmental conditions (Bewley, 1997). Seed dormancy exists widely in angiosperms and is usually divided into physiological dormancy, morphological dormancy, morphophysiological dormancy, physical dormancy, and combinational dormancy (Baskin and Baskin, 2004). It is well known that seed dormancy is considered an adaptive characteristic, contributes to seedling establishment in time and space (Baskin and Baskin, 1998; Baskin and Baskin, 2004; Bewley and Black, 1994), and further ensures the existence of plant populations in unpredictable environments (Baskin and Baskin, 1998). Despite its significance, seed dormancy has been considered “one of the least understood phenomena in seed biology” (Finch-Savage and Leubner-Metzger, 2006) and continues to be a research hotspot in seed biology. Therefore, understanding the mechanisms of seed dormancy release is important for seed biology investigation.

*S. glauca* is one of the important halophytes in China, mainly distributed in the saline-alkaline soils located in beaches, wastelands, field margins, and canal banks (Delectis Florae Reipublicae Popularis Sinicae Agendae Academiae Sinicae Edita, 1979). This species possesses important economic and ecological values due to its edible traits, high oil content, and salt tolerance (Zhao et al., 2002; Duan et al., 2018). Previous studies showed that *S. glauca* produced dimorphic seeds on the same plant, including brown seeds with non-dormant characteristics and black seeds with intermediate physiological dormancy (Wang et al., 2020). The irregularity of seedling emergence resulting from seed dormancy greatly restricts its extensive application in artificial cultivation and the ecological restoration of saline-alkaline soils. Wang et al. (2020) found that low concentrations (0.1 mM and 1.0 mM) of gibberellic acid 3 (GA_3_) significantly promoted the germination of *S. glauca* black seeds. Similarly, the stimulative effects of GA_3_ on the germination of black seeds were also presented in *S. acuminata* (Wang et al., 2012) and *S. aralocaspica* (Wang et al., 2008). However, the physiological and molecular mechanisms of GA_3_-induced dormancy release and the germination of black seeds in the genera *Suaeda* remain unclear.

Many studies have shown that gibberellins (GAs) promote seed germination by inducing hydrolytic enzymes that reduce the barriers of the endosperm and seed coat (Groot et al., 1988; Leubner-Metzger et al., 1996; Finch-Savage and Leubner-Metzger, 2006). GAs also enhance the growth potential of the embryo by antagonizing ABA activity (Finch-Savage and Leubner-Metzger, 2006). The molecular mechanisms of GA_3_-induced seed dormancy release have been explored using transcriptomic and/or metabolomic analysis in *Fraxinus hupehensis* (Song et al., 2019), *Leymus chinensis* (Li et al., 2021), *Phyllostachys edulis* (Li et al., 2022), *Panax notoginseng* (Ge et al., 2023), and *Zea mays* (Han et al., 2023). However, the mechanism of seed dormancy release in different plants might be species-dependent (Chibani et al., 2006; Lee et al., 2006; Pawłowski, 2007; Pawłowski, 2009; Deng et al., 2016). Moreover, Hauvermale et al. (2015) found that genes related to GA_3_ response elements were differentially expressed in *Arabidopsis* seeds with varying dormancy levels. Thus, the regulatory mechanisms of GA_3_ on dormancy release in *S. glauca* black seeds warrant further investigation.

In this study, *S. glauca* black seeds were used as experimental materials, and the transcriptome differences between seed samples at different germination stages, with and without GA_3_ treatment, were analyzed to identify important metabolism pathways and candidate genes related to GA_3_-induced dormancy release. Our findings aim to provide new insights into how GA_3_ promotes seed dormancy release and extend our knowledge of seed dormancy and germination.

## Materials and methods

### Plant materials and seed treatments

Dimorphic seeds (brown and black) of *S. glauca* were collected from plants in a natural population at the Yingchengzi coastal saline beach (121.36^°^ E, 38.99^°^ N) located in Dalian City, Liaoning Province, China, on 25 October 2021. All seeds were collected from at least 100 plants and dried for seven days at room temperature. The dimorphic seeds were separated according to their phenotypic characteristics (Wang et al., 2020). For this study, black seeds with physiological dormancy characteristics were used.

### Seed germination assays and seed sample collection

In order to identify the effects of GA_3_ on the dormancy release of *S. glauca* black seeds, 50 seeds were placed in 9-cm-diameter petri dishes containing two layers of Whatman No. 1 filter paper (Shanghai Root Biotechnology Co., Ltd., Shanghai) and wetted using 10 mL of different solutions: GA_3_ solution (GA_3_), distilled water (DW), and phosphate buffer solution (PBS). Based on the solubility of GA_3_ (Solarbio Science and Technology Co., Ltd., Beijing), it was dissolved in 0.01 mol/L phosphate buffer solution for the germination assays, and 1.0 mmol/L GA_3_ of optimal concentration was used in the present experiment, according to Wang et al. (2020). The germination experiments were carried out in an incubator (MGC-100P, Shanghai Yiheng Scientific Instrument Co., Ltd., Shanghai) at 20°C/10°C with 12 h light and 12 h dark conditions. Fluorescent lamps were used as the light source, and the light intensity was 150 μmol m^-2^s^-1^. Germination was observed daily for 30 days, and seeds with a visible radicle >2 mm were considered to have germinated. The germination rate was then calculated. All germination experiments consisted of three biological replicates, with each replicate having 50 seeds.

**Figure 1:**
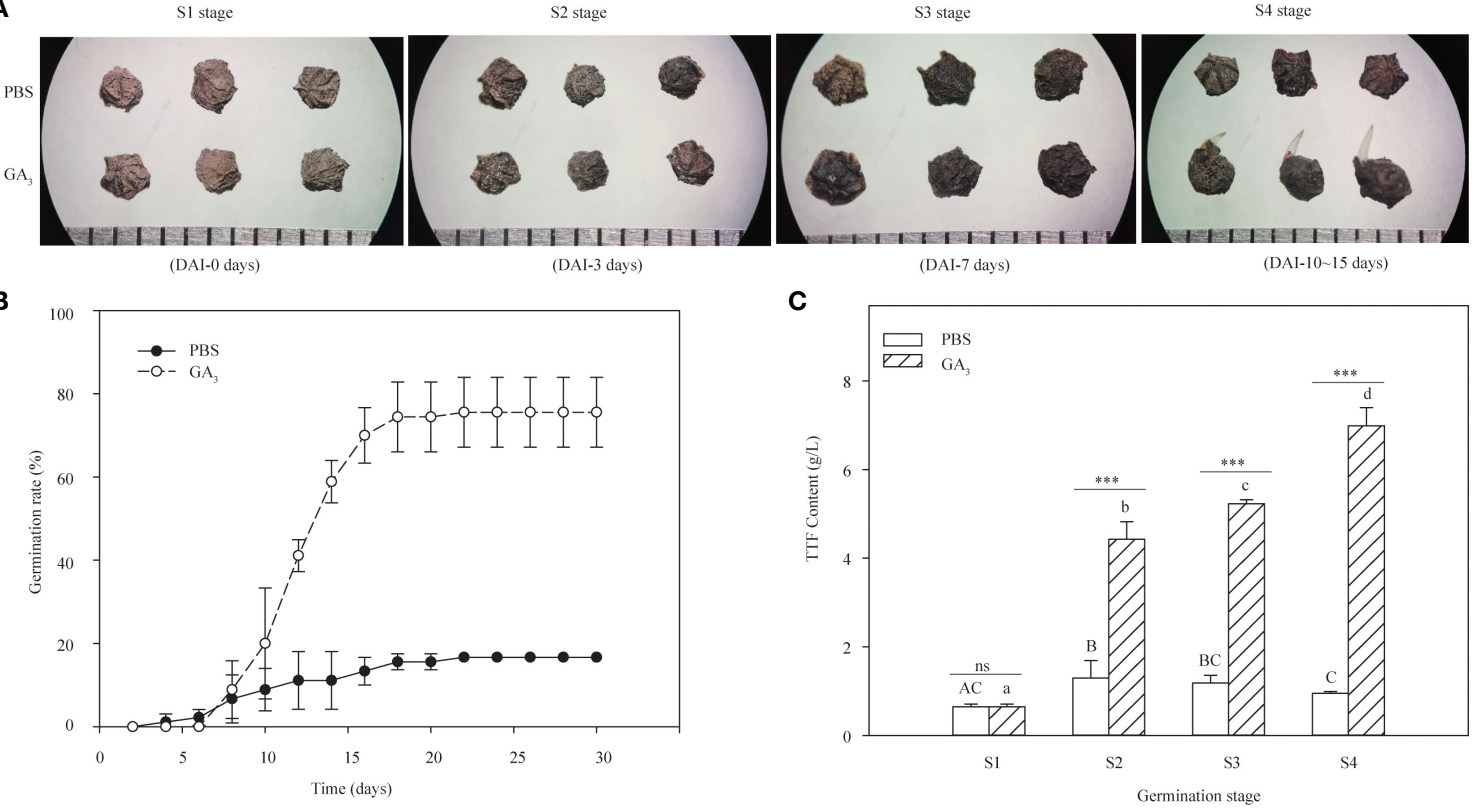
Effects of GA_3_ on the germination rate and seed vigor of *S. glauca* black seeds. **(A)** Phenotypic differences of seeds at different germination stages treated with and without GA_3_. **(B)** The germination rate of seeds with or without GA_3_ treatment at 20°C/10°C for 30 days. **(C)** The seed vigor with and without GA_3_ treatment at different germination stages. PBS: seeds germinated in the phosphate buffer solutions; GA_3_: seeds germinated in the GA_3_ solutions. S1: dry seed stage; S2: imbibition stage; S3: initial stage of seed germination; S4: radicle emergence stage. Different upper-case letters indicate significant differences for seeds with PBS treatment across different germination stages, according to the LSD test at *P*< 0.05. Different lower-case letters indicate significant differences for seeds with GA_3_ treatment across different germination stages, according to the LSD test at *P*< 0.05. “ns” and “***” represent no difference and a highly significant difference according to the t-test, respectively. Vertical bars indicate means ± SD (n=3).

Based on the germination dynamics of black seeds presented in GA_3_ solutions (**Figure 1**), the germination process was divided into four stages: the dry seed stage (S1), the imbibition stage (S2), the initial stage of seed germination (S3), and the radicle emergence stage (S4), which corresponds to 0 days, 3 days, 7 days, and 10–15 days of the germination experiment, respectively.

The preliminary experiment revealed that 0.01 mol/L phosphate buffer solutions had no significant difference in seed germination rate compared with distilled water (**Supplementary Figure S1**). Taken together, seed samples (30 seeds per sample) collected from GA_3_ treatment (GA_3_ group) at S1, S2, S3, and S4 stages, respectively, were used as experimental materials; meanwhile, seed samples collected from PBS treatment (PBS group) were used as controls. The collected seed samples were immediately frozen in liquid nitrogen and stored at −80°C for transcriptome analysis and physiological indicator determination. Of them, seed samples collected from the S1 and S4 stages were used for transcriptome analysis. All seed samples consisted of three biological replicates at each germination stage.

### RNA isolation, cDNA library construction, and transcriptome sequencing

Total RNA extraction from each sample was performed using TRIzol^®^ Reagent (Plant RNA purification reagent for plant tissue) (Invitrogen, Carlsbard, CA, USA) according to the manufacturer’s instructions, and genomic DNA was removed using DNase I (TaKaRa). The quality, purity, and integrity of total RNA were determined using 1% agarose electrophoresis with the Nanodrop 2000 (NanoDrop Thermo Scientific, Wilmington, DE, USA) and Agilent 2100 Bioanalyser (Agilent Technologies, Inc., Santa Clara, CA, USA), respectively. Only high-quality RNA samples (OD_260/280_ = 1.8~2.2, OD_260/230_ ≥ 2.0, RIN ≥ 8.0, and >2μg) were used to construct the sequencing library. RNA purification, reverse transcription, library construction, and sequencing were performed at Shanghai Majorbio Bio-Pharm Biotechnology Co., Ltd. (Shanghai, China) according to the manufacturer’s instructions (Illumina, San Diego, CA, USA). Finally, a total of nine RNA-seq libraries of *S. glauca* black seeds at two critical germination stages with or without GA_3_ treatment were sequenced on the Illumina sequencing platform (Hiseq X ten) (Illumina, San Diego, CA, USA), and 300 bp paired-end reads were generated for each sample.

### *De novo* assembly and functional annotation of unigenes

After sequencing, the raw paired end reads were trimmed and quality controlled using SeqPrep (https://github.com/jstjohn/SeqPrep) and Sickle (https://github.com/najoshi/sickle) with default parameters. All further analyses in this study were based on high-quality, clean data. *De novo* assembly of clean reads was performed using Trinity software (version 2.8.5) (http://trinityrnaseq.sourceforge.net/). According to the similarity and length of the sequence, the longest transcript was selected as a single gene for subsequent analysis. The assembled unigenes were annotated using the non-redundant protein (NR) (https://www.ncbi.nlm.nih.gov/) and eggNOG (http://eggnogdb.embl.de/), SwissProt (http://www.expasy.ch/sprot/), KEGG (http://www.genome.jp/kegg/), GO (http://www.geneontology.org), and Pfam (http://pfam.xfam.org/) databases.

### Differentially expressed unigenes analysis

The expression level of each unigene was calculated according to the fragments per kilobase of exon per million mapped reads (FPKM) method. Differentially expressed genes (DEGs) were identified using the R statistical package software DESeq2 (version 1.24.0) (http://www.bioconductor.org/packages/stats/bioc/DESeq2/) with a threshold of |log_2_ (fold change)(FC)| >2 with FPKM >0, and Benjamini and Hochberg (BH) corrected *P*-value <0.05. Hierarchical cluster analysis of DEGs was performed to explore gene expression patterns. KEGG pathway enrichment analysis of DEGs was performed using the KEGG database (http://www.kegg.jp/), with BH corrected *P*-value <0.05 as the threshold and rich factor.

### Physiological indicator measurement

Seed vigor was assessed using a quantitative 2,3,5-triphenyltetrazolium chloride (TTC) assay (Ma et al., 2019). Thirty embryos were collected from seed samples with a scalpel, weighed, and then ground into powder with liquid nitrogen. The content of triphenyl tetrazolium formazan (TTF) was determined using a spectrophotometer (UV-1100, Shanghai Macylab Instrument Co., Ltd., Shanghai) at 484 nm. All experiments consisted of three replicates.

The superoxide anion (O_2_^–^) and hydrogen peroxide (H_2_O_2_) contents were measured using a superoxide anion assay kit (Cat#BC1290, Solarbio Science and Technology Co., Ltd., Beijing) and a hydrogen peroxide assay kit (Cat#BC3590, Solarbio Science and Technology Co., Ltd., Beijing), respectively. Each experiment consisted of three replicates. The extraction and determination were performed according to the manufacturer’s instructions. Briefly, 100 mg of seed samples were supplemented with 1 mL of extraction solution, homogenized in an ice bath, and then centrifuged at 8000 rpm for 20 min at 4°C. The content of O_2_^–^ and H_2_O_2_ was determined using a spectrophotometer (UV-1100, Shanghai Macylab Instrument Co., Ltd., Shanghai) at 530 nm and 415 nm, respectively.

The determination of soluble sugar content and starch content was performed as described by Liu et al. (2020), with minor modifications. In total, 100 mg of seed samples were homogenized with liquid nitrogen in a pre-cooling mortar and pestle and put into a 10 mL test tube. Next, 4 mL of 80% ethanol was added, heated, and continually stirred in a water bath at 80°C for 40 min. After cooling, the extracting solutions were centrifuged at 8000 rpm for 20 min at 25°C, and the supernatant was collected. The residues were resuspended in 2 mL of 80% ethanol, and the extracting process was repeated two times. All supernatants were then combined, 10 mg of active carbon was added, and the mixture was boiled in a water bath at 80°C for 30 min. The extraction solutions were filtered and then diluted to 10 mL with 80% ethanol for soluble sugar determination.

The residues of the ethanol extract were saved for starch analysis. Firstly, the residues were transferred into a 50-mL volumetric flask, and 10 mL of distilled water was added. The mixture was then boiled in a water bath for 30 min. Secondly, 2 mL of 72% perchloric acid was added and used to extract for 15 min. After cooling, the extracting solutions were filtered and diluted to 50 mL using distilled water. The content of soluble sugar and starch was determined using the anthrone colorimetry method. A standard response curve was prepared with a known concentration of glucose or soluble starch using the same method as described above. All experiments consisted of three biological replicates. The results are expressed as mg g-1 FW.

### Quantitative real-time PCR analysis

To verify the accuracy of transcriptomic data, 15 DEGs involved in the dormancy release of *S. glauca* black seeds were selected for qRT-PCR analysis, and *SgActin* was used as the internal reference gene. The primers were designed based on the sequence of candidate genes using the software Primer 5.0. The primers were synthesized by Sangon Biotech (Shanghai) Co., Ltd. (Shanghai, China) and listed in **Supplementary Table S9**. The RNA extracted from nine seed samples was used to synthesize cDNA using a PrimeScript^®^RT Reagent Kit with gDNA Eraser (Perfect Real Time) (TaKaRa) according to the manufacturer’s instructions. The qRT-PCR reaction system contained 10 μL of 2×*PerfectStart* ^®^Green qPCR SuperMix (TransGen), 0.4 μL of forward primer, 0.4 μL of reverse primer, 1 μL of cDNA, and 8.2 μL of ddH_2_O. The qRT-PCR reaction was performed using a Thermal Cycler Dice Real Time System TP-800 instrument (TaKaRa, Japan). Each sample possessed three biological replicates, and each replicate contained three technical replicates. The relative expression level of the candidate gene was calculated using the 2^−ΔΔCt^ method. The normalized values of relative expression and FPKM value were calculated using log_2_ fold change measurements, and the correlation between the RNA-Seq and qRT-PCR results was analyzed using these values.

### Statistical analysis of data

All data are the result of three biological replicates and shown as means ± standard deviation (SD) (n = 3). All analyses were completed using Microsoft Excel 2013, SigmaPlot 12.5, and SPSS 20.0 software. The changes in TTF content, starch and soluble sugar content, and endogenous H_2_O_2_ and O_2_^–^ content during GA_3_-induced dormancy release were analyzed using a one-way ANOVA followed by the LSD multiple comparisons test at *P* < 0.05. An independent t-test was also used to determine the difference in different physiological indicators between the GA_3_ group and the PBS group at *P* < 0.05.

## Results

### Effect of GA_3_ treatment on germination rate and seed vigor

The effects of exogenous GA_3_ on the germination of black seeds were tested. Approximately 16.7% germination was obtained for seeds treated without GA_3_ (PBS group) after 30 days of incubation at 20°C/10°C with 12 h photoperiod conditions, while about 75.56 ± 8.39% germination rate was achieved in the presence of 1 mM GA_3_ conditions (GA_3_ group) (**Figures 1A, B**).

To evaluate the effects of exogenous GA_3_ on seed vigor, changes in the TTF content of black seeds at different germination stages treated with or without 1 mM GA_3_ were assessed. The results showed that the TTF content of seeds treated with GA_3_ increased significantly with germination progression (F = 255.75, *P* < 0.001) and exhibited the highest content at the S4 stage (radicle ermergence stage), while the TTF content of seeds treated without GA_3_ increased first with seed imbibition and significantly decreased thereafter (**Figure 1C**). Meanwhile, significant differences were presented in the TTF content between the GA_3_ group and the PBS group at the S2 (imbibition stage), S3 (initial stage of seed germination), and S4 stages, except for the S1 stage (dry seed stage). Specifically, the TTF content of the seeds treated with GA_3_ was significantly higher than that of the seeds treated without GA_3_ at the S2, S3, and S4 stages (*P* < 0.001, **Figure 1C**). Taken together, these results indicate that exogenous GA_3_ can significantly enhance the seed’s vigor and further improve its germination capacity.

### RNA-seq *de novo* assembly and function annotation during GA_3_-induced dormancy release

To identify the change in gene expression under GA_3_-induced seed dormancy release, seed samples at different germination stages treated with and without GA_3_ [B0_1, B0_2, and B0_3; Bg_1, Bg_2, and Bg_3; Pg_1, Pg_2, and Pg_3] were used to construct RNA-Seq libraries. A total of nine cDNA libraries were sequenced using the Illumina Hiseq platform at Shanghai Majorbio Bio-Pharm Technology Co., Ltd. A total of 58.05 Gb of clean data were obtained after removing low-quality reads and adapter sequences. The Q30 values of nine samples exceeded 93.05%, and the GC content ranged from 43.73% to 44.90% (**Supplementary Table S1**). A total of 128,280 unigenes were obtained after *de novo* assembly using Trinity software (**Supplementary Table S2**). The average length of unigenes was 736.75 bp, and the length of N50 was 1130 bp. The length of 25,030 (19.51%) unigenes was over 1000 bp. The Pearson correlation coefficient values (R^2^) of three biological replicates under the same treatment were higher than 0.91 (**Supplementary Table S3**), which suggests that the biological replicates were highly correlated and the transcriptome data was satisfactory. The number of unigenes annotated in the NR database was 63,204 (49.27%), 65,535 (51.09%) were obtained in the EggNOG database, 54,715 (42.65%) were obtained in the SwissProt database, 43,930 (34.25%) were obtained in the KEGG database, 49,974 (38.96%) were obtained in the GO database, and 58,278 (45.43%) were obtained in the Pfam database (**Supplementary Table S2**).

### Comparative analysis of DEGs

**Figure 2:**
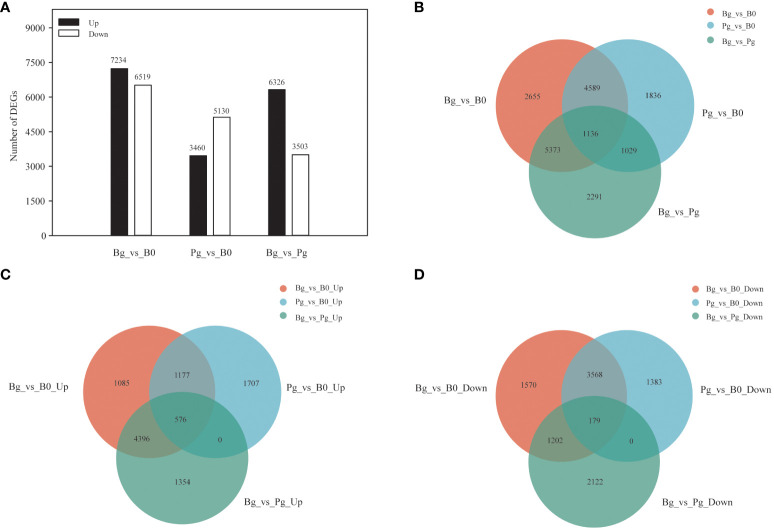
Multivariate statistical analysis of transcriptome data. **(A)** The numbers of up-regulated and down-regulated DEGs in different comparison groups. **(B)** Venn diagram showing the overlapping and unique DEGs in the comparison groups “Bg_vs_B0,” “Pg_vs_B0,” and “Bg_vs_Pg.” **(C)** Venn diagram showing the overlapping up-regulated DEGs in three comparison groups. **(D)** Venn diagram showing the overlapping down-regulated DEGs in three comparison groups. DEGs, differentially expressed genes; UP, up-regulated DEGs; DOWN, down-regulated DEGs.

To investigate the potential regulatory mechanism of exogenous GA_3_-induced dormancy release of *S. glauca* black seeds, three comparison groups, including black seeds soaked in 1 mM GA_3_ solutions for germination (Bg) vs. dry dormant black seeds (B0) (Bg_vs_B0 group), black seeds soaked in PBS solutions for germination (Pg) vs. dry dormant black seeds (B0) (Pg_vs_B0 group), and black seeds soaked in 1 mM GA_3_ solutions for germination (Bg) vs. black seeds soaked in PBS solutions for germination (Pg) (Bg_vs_Pg group), were constructed and used for subsequent analysis. We adopted the criteria |log_2_FC| >2.0 with FPKM >0 and BH adjusted *P <*0.05 to identify differentially expressed unigenes (DEGs) in different comparison groups. A total of 13,753 (7234 up- and 6519 down-regulated), 8590 (3460 up- and 5130 down-regulated), and 9829 (6326 up- and 3503 down-regulated) DEGs were presented in the Bg_vs_B0 group, the Pg_vs_B0 group, and the Bg_vs_Pg group, respectively (**Figure 2A**). In addition, Venn diagram analysis was obtained to display the overlap among different comparison groups. A total of 1136 DEGs were co-expressed among three comparison groups (**Figure 2B**) (**Supplementary Table S4**). Of them, 576 DEGs were up-regulated (**Figure 2C**), and 179 DEGs were down-regulated (**Figure 2D**). The results indicate that these co-expressed DEGs may play important roles in GA_3_-induced dormancy release in *S. glauca* black seeds.

### Kyoto encyclopedia of genes and genomes analysis of DEGs

**Figure 3:**
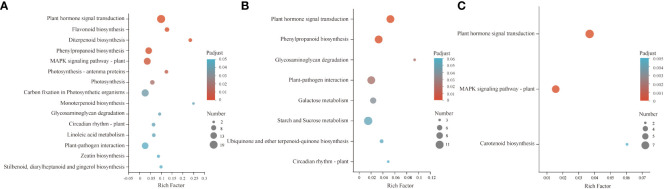
Kyoto Encyclopedia of Genes and Genomes (KEGG) pathway enrichment analysis of DEGs. **(A)** the enriched pathways in 1136 DEGs. **(B)** the enriched pathways in 576 up-DEGs. **(C)** the enriched pathways in 179 down-DEGs. The colors are shaded according to the *P* adjust-value level, as shown in the color bars, gradually from low (blue green) to high (red). The size of the circle indicates the number of DEGs, from small (less) to big (more).

KEGG enrichment analysis was used to evaluate the potential functions of related DEGs during the GA_3_-induced dormancy release process. The results revealed that 1136 co-expressed DEGs in three comparison groups were mainly involved in plant hormone signal transduction, flavonoid biosynthesis, diterpenoid biosynthesis, phenylpropanoid biosynthesis, and MAPK signaling pathway-plant pathways (**Figure 3A**). Of them, the up-regulated 576 DEGs were mainly enriched in plant hormone signal transduction, phenylpropanoid biosynthesis, galactose metabolism, and starch and sucrose metabolism pathways (**Figure 3B**). In contrast, the down-regulated 179 DEGs were enriched in plant hormone signal transduction, the MAPK signaling pathway-plant, and carotenoid biosynthesis (**Figure 3C**). Therefore, changes in plant hormone signal transduction, diterpenoid biosynthesis, carotenoid biosynthesis, flavonoid biosynthesis, phenylpropanoid biosynthesis, and energy metabolism pathways may play important roles in GA_3_-induced dormancy release in *S. glauca* black seeds.

### DEGs related to plant hormone signal transduction, diterpenoid, and carotenoid biosynthesis during GA_3_-induced dormancy release

**Figure 4:**
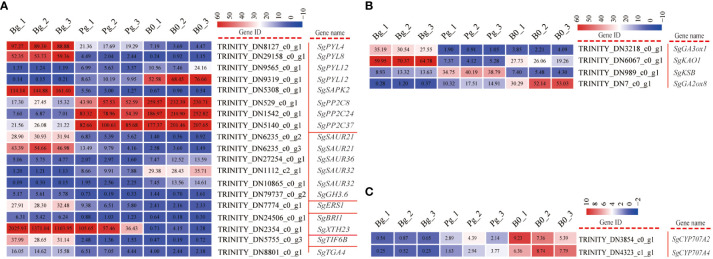
DEGs involved in plant hormone signaling transduction and ABA and GA metabolism pathways in *S. glauca* black seeds during GA_3_-induced seed dormancy release. **(A)** Plant hormone signal transduction pathway and expression of related genes. **(B)** GA metabolism pathway and expression of related genes. **(C)** ABA metabolism pathway and expression of related genes. Expression levels ranging from red to blue indicate high to low expression for genes, respectively. Gene ID and name are shown by the legend on the right. The values presented in the box indicate the absolute FPKM value of each sample in each group.

A transcriptomic database revealed 19 genes involved in auxin (AUX), abscisic acid (ABA), ethylene (ETH), brassinosteroid (BR), jasmonic acid (JA), and salicylic acid (SA) signaling pathways were significantly expressed during GA_3_-induced dormancy release (**Supplementary Table S5**). Of them, AUX and ABA were major constituents of plant hormone signal pathways (**Figure 4A**), including five auxin responsive protein (SAUR), one indole-3-acetic acid-amido-synthetase (GH3), four abscisic acid receptors (PYR/PYL), three protein phosphatase 2 C (PP2C), and one serine/threonine-protein kinase (SAPK2). In the case of the AUX signal pathway, *SgGH3.6* (TRINITY_DN79737_c0_g2) exhibited an increasing trend in expression levels, while 5 *SgSAURs* (TRINITY_DN10865_c0_g1, TRINITY_DN6235_c0_g2, TRINITY_DN6235_c0_g3, TRINITY_DN27254_c0_g1, and TRINITY_DN1112_c0_g1) presented different expression profiles (**Figure 4A**). The major constituents involved in the ABA signal pathway were the abscisic acid receptor (PYR/PYL) and protein phosphatase 2C (PP2C). Among them, all of the *SgPP2Cs* genes (TRINITY_DN1542_c0_g1, TRINITY_DN5140_c0_g1, and TRINITY_DN529_c0_g1) exhibited a decreasing trend, while the *SgPYLs* genes were observed to be differentially regulated. Interestingly, the log_2_ FC of *SgPP2C24*, *SgPP2C37*, and *SgPP2C08* in the “Bg_vs_Pg group” was -3.11, -1.75, and -1.15, respectively (**Supplementary Table S5**).

In addition, a total of six genes associated with diterpenoid (**Figure 4B**) and carotenoid (**Figure 4C**) biosynthesis were differentially expressed during GA_3_-induced dormancy release. Among them, *SgGA3ox1* (TRINITY_DN3218_c0_g1) and *SgKAO1* (TRINITY_DN6067_c0_g1) genes exhibited an increasing trend, while *SgGA2ox8* (TRINITY_DN7_c0_g1) exhibited a decreasing trend. Especially, the log_2_ FC of *SgGA3ox1* in “Bg_vs_B0” and “Bg_vs_Pg” was 3.36 and 4.83, respectively (**Supplementary Table S5**). Meanwhile, *SgCYP707A2* (TRINITY_DN3854_c0_g1) and *SgCYP707A4* (TRINITY_DN4323_c0_g1) exhibited a decreasing trend and displayed the highest expression level in dry dormant seeds (**Figure 4C**). These results indicate that although the genes related to ABA degradation are inhibited under GA_3_ treatment, the genes related to GA synthesis are activated, and the degradation of GA is also inhibited, enhancing the GA/ABA ratio.

### DEGs related to phenylpropanoid and flavonoid biosynthesis during GA_3_-induced dormancy release

**Figure 5:**
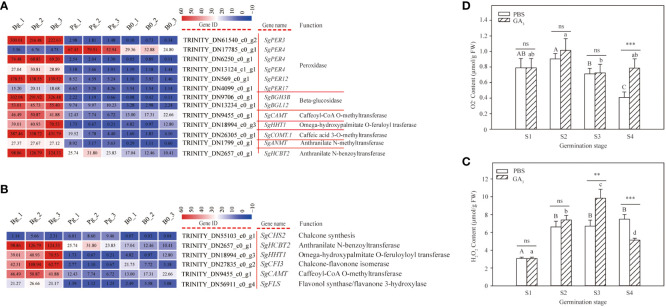
DEGs involved in phenylpropanoid and flavonoid biosynthesis pathways and H_2_O_2_ and O_2_^-^ contents in *S. glauca* black seeds during GA_3_-induced seed dormancy release. **(A)** Phenylopropanoid biosynthesis pathway and expression of related metabolic enzyme genes. **(B)** Flavonoid biosynthesis pathway and expression of related metabolic enzyme genes. **(C)** H_2_O_2_ content. **(D)** O_2_^-^ content. Expression levels ranging from red to blue indicate high to low expression for genes, respectively. Gene ID, name, and function are shown by the legend on the right. The values presented in the box indicate the absolute FPKM value of each sample in each group. PBS: seeds germinated in the phosphate buffer solutions; GA_3_: seeds germinated in the GA_3_ solutions. S1: dry seed stage; S2: imbibition stage; S3: initial stage of seed germination; S4: radicle emergence stage. Different upper-case letters indicate significant differences for seeds with PBS treatment across different germination stages, according to the LSD test at *P*< 0.05. Different lower-case letters indicate significant differences for seeds with GA_3_ treatment across different germination stages, according to the LSD test at *P*< 0.05. “ns,” “**,” and “***” represent no difference, significant difference, or highly significant difference according to the t-test, respectively. Vertical bars indicate means ± SD (n=3).

The DEGs related to phenylpropanoid and flavonoid biosynthesis pathways were examined in this study to determine their function during GA_3_-induced dormancy release (**Supplementary Table S6**). A total of 13 DEGs (12 up-regulated, 1 down-regulated) were enriched in the phenylpropanoid biosynthesis pathway (**Figure 5A**). Of them, the major constituents involved in the phenylpropanoid biosynthesis pathway were peroxidases (PERs). Five out of six genes encoding peroxidase, including *SgPER3* (TRINITY_DN61540_c0_g2), *SgPER4* (TRINITY_DN6250_c0_g1), *SgPER4* (TRINITY_DN13124_c1_g1), *SgPER12* (TRINITY_DN569_c0_g1), and *SgPER17* (TRINITY_DN4099_c0_g1), exhibited an increasing trend, while *SgPER4* (TRINITY_DN17785_c0_g1) presented an opposite change. In addition, the expression levels of *SgBGH3B* (TRINITY_DN9706_c0_g1), *SgBGL12* (TRINITY_DN13234_c0_g1), *SgCAMT* (TRINITY_DN9455_c0_g1), *SgHHT1* (TRINITY_DN18994_c0_g3), *SgCOMT1* (TRINITY_DN26305_c0_g1), *SgANMT* (TRINITY_DN1799_c0_g1), and *SgHCBT2* (TRINITY_DN2657_c0_g1) also exhibited an increasing trend. These results indicate that the phenylpropanoid biosynthesis pathway plays important roles in the dormancy release of *S. glauca* black seeds, especially peroxidase, which has important functions for seed dormancy release.

Flavonoid is one of the important phenylpropanoid-derived compounds; a total of six DEGs were enriched in the flavonoid biosynthesis pathway. As shown in **Figure 5B**, most of the DEGs exhibited an increasing trend during GA_3_-induced dormancy release. Among them, the expression levels of *SgHCBT2* (TRINITY_DN2657_c0_g1), *SgHHT1* (TRINITY_DN18994_c0_g3), *SgCFI3* (TRINITY_DN27835_c0_g2), *SgCAMT* (TRINITY_DN9455_c0_g1), and *SgFLS* (TRINITY_DN56911_c0_g4) were significantly higher in the GA_3_-treated seeds (**Figure 5B**). Especially, the log_2_ FC of *SgCFI3* (TRINITY_DN27835_c0_g2), *SgHHT1* (TRINITY_DN18994_c0_g3), *SgCAMT* (TRINITY_DN9455_c0_g1), and *SgFLS* (TRINITY_DN56911_c0_g4) in “Bg_vs_B0 group” was 2.91, 3.08, 1.46, and 2.67, respectively, while the corresponding value in “Pg_vs_B0 group” was -2.77, -2.87, -1.03, and -1.61, respectively (**Supplementary Table S6**). These results indicate that flavonoid biosynthesis is a positive regulatory pathway for GA_3_-induced dormancy release.

It is well known that the secondary metabolites produced through the phenylpropanoid and flavonoid biosynthesis pathways act as effective reactive oxygen species (ROS) scavenging compounds. To study the role of ROS during GA_3_-induced dormancy release, the contents of H_2_O_2_ and O_2_^–^ were measured. In our study, the H_2_O_2_ content of seeds treated with GA_3_ increased with seed imbibition, exhibited the highest content at the S3 stage, and significantly decreased thereafter (**Figure 5C**). In contrast, the H_2_O_2_ content of seeds treated without GA_3_ also increased with seed imbibition, but no changes thereafter were observed. Meanwhile, there was no significant difference presented in H_2_O_2_ contents between the GA_3_ group and the PBS group at the S2 stage, but a significant difference was observed at the S3 and S4 stages (**Figure 5C**). Of them, the H_2_O_2_ content of the seeds with GA_3_ treatment was significantly higher than that of the seeds without GA_3_ treatment at the S3 stage but showed the opposite change at the S4 stage (**Figure 5C**). These results indicate that H_2_O_2_ plays an important role at the initial stage of seed germination and promotes seed dormancy release. As far as O_2_^-^ content is concerned, the O_2_^–^ content of seeds treated without GA_3_ declined sharply from the S1 stage to the S4 stage, but no significant change was observed in seeds treated with GA_3_ (F=3.208, *P*=0.083) (**Figure 5D**). Meanwhile, there was no significant difference presented in O_2_^–^ content between the GA_3_ group and the PBS group at the S1, S2, and S3 stages, but the O_2_^–^ content of the seeds treated with GA_3_ was significantly higher than that of the seeds treated without GA_3_ (**Figure 5D**) at the S4 stage. These results indicate that O_2_^–^ may play an important role during the emergence and growth of the radicle.

### DEGs related to carbohydrate metabolism during GA_3_-induced dormancy release

**Figure 6:**
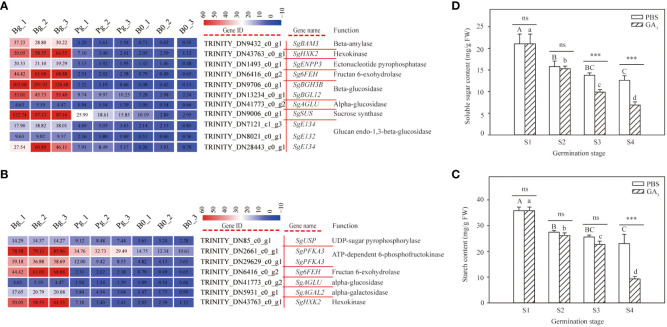
DEGs involved in carbohydrate metabolism pathways and starch and soluble sugar contents in *S. glauca* black seeds during GA_3_-induced seed dormancy release. **(A)** Starch and sucrose metabolism pathways and expression of related metabolic enzyme genes. **(B)** Galactose metabolism pathway and expression of related metabolic enzyme genes. **(C)** Starch content. **(D)** Soluble sugar content. Expression levels ranging from red to blue indicate high to low expression for genes, respectively. Gene ID, name, and function are shown by the legend on the right. The values presented in the box indicate the absolute FPKM value of each sample in each group. PBS: seeds germinated in the phosphate buffer solutions; GA_3_: seeds germinated in the GA_3_ solutions. S1: dry seed stage; S2: imbibition stage; S3: initial stage of seed germination; S4: radicle emergence stage. Different upper-case letters indicate significant differences for seeds with PBS treatment across different germination stages, according to the LSD test at *P*< 0.05. Different lower-case letters indicate significant differences for seeds with GA_3_ treatment across different germination stages, according to the LSD test at *P*< 0.05. “ns” and “***” represent no difference and a highly significant difference according to the t-test, respectively. Vertical bars indicate means ± SD (n=3).

The KEGG enrichment analysis suggested that the DEGs involved in carbohydrate metabolism were enriched in the starch and sucrose metabolism pathways and the galactose metabolism pathway during GA_3_-induced dormancy release. Of them, 11 DEGs and seven DEGs were enriched in the starch and sucrose metabolism pathways and the galactose metabolism pathway, respectively (**Supplementary Table S7**). Cluster analysis revealed that all DEGs involved in carbohydrate metabolism exhibited an increasing trend (**Figures 6A, B**). Especially, the log_2_ FC of *SgBAM3, SgAGLU*, and *SgHXK2* in “Bg_vs_Pg” was 4.68, 1.77, and 3.86, respectively (**Supplementary Table S7**). These results indicate that the carbohydrate metabolism pathway plays an important role during GA_3_-induced dormancy release.

In this study, all genes involved in carbohydrate metabolic enzymes were up-regulated in the seeds treated with GA_3_ during the germination process. To investigate the role of carbohydrate metabolism in seed germination, we determined the starch and soluble sugar contents of seeds with or without GA_3_ treatment at different germination stages. The results showed that the starch content of the seeds treated with or without GA_3_ significantly decreased with the germination process (**Figure 6C**). However, there was no significant difference presented in starch content between the GA_3_ group and the PBS group at the S1, S2, and S3 stages, whereas the starch content of the seeds treated with GA_3_ was significantly lower than that of the seeds treated without GA_3_ at the S4 stage (**Figure 6C**). These results indicate that GA_3_ treatment accelerates the degradation of starch. A similar trend was also observed in soluble sugar content (**Figure 6D**); no significant difference was presented in sugar content between the GA_3_ group and the PBS group at the S1 and S2 stages, while the soluble sugar content of the seeds treated with GA_3_ was significantly lower than that treated without GA_3_ at the S3 and S4 stages (**Figure 6D**). These results indicate that the soluble sugar content significantly decreases and is consumed for radicle growth during GA_3_-induced dormancy release.

### Validation of RNA-seq data by qRT-PCR

**Figure 7:**
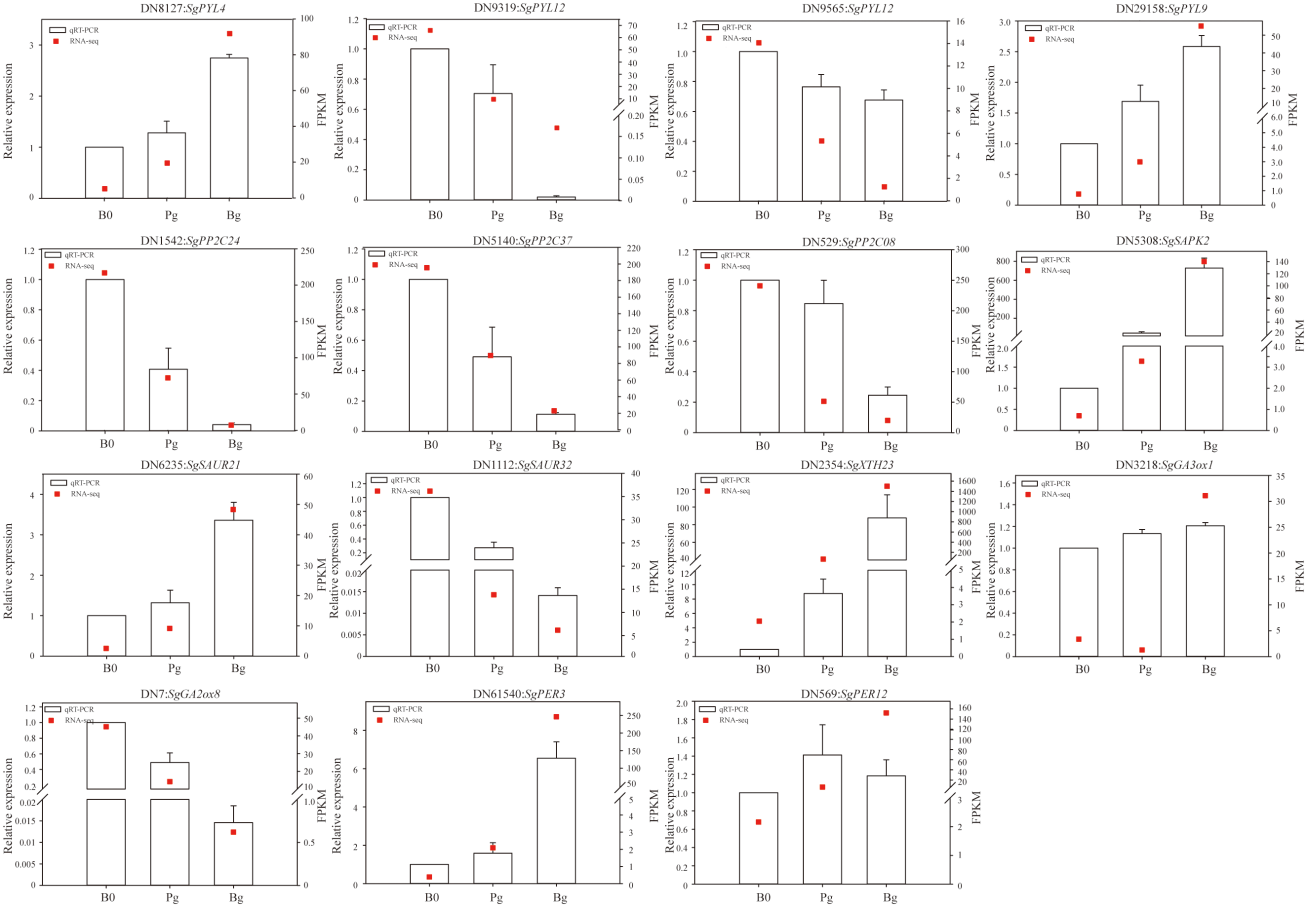
The qRT-PCR validation of 15 candidate DEGs. The relative expression levels of qRT-PCR were obtained using actin as a standard. DN8127:*SgPYL4* (abscisic acid receptor), DN9319:*SgPYL12* (abscisic acid receptor), DN9565:*SgPYL12* (abscisic acid receptor), DN29158:*SgPYL9* (abscisic acid receptor), DN1542:*SgPP2C24* (protein phosphatase 2C), DN5140:*SgPP2C37* (protein phosphatase 2C), DN529:*SgPP2C08* (protein phosphatase 2C), DN5308:*SgSAPK2* (serine/threonine-protein kinase), DN6235:*SgSAUR21* (small auxin up RNA), DN1112:*SgSAUR34* (small auxin up RNA), DN2354:*SgXTH23* (xyloglucan endotransglucosylase/hydrolase), DN7:*SgGA2ox8* (gibberellin 2 oxidase), DN3218:*SgGA3ox1* (gibberellin 3 oxidase), DN61540:*SgPER3* (peroxidase), DN569:*SgPER12* (peroxidase).

To further confirm the reliability of the RNA-seq results, 15 DEGs enriched in KEGG pathways during GA_3_-induced dormancy release were selected randomly for qRT-PCR analysis (**Supplementary Table S8**). The specific primers for these DEGs were designed by Primer 5.0 software (**Supplementary Table S9**). The qRT-PCR results indicated that the expression trends of 15 genes (100%) were consistent with the transcriptome data in the “Bg_vs_B0” group (**Figure 7**), with only the difference of the absolute fold changes of gene expression (**Supplementary Table S8**). However, the qRT-PCR results showed that *SgGA3ox1* (TRINITY_DN3218_c0_g1) was up-regulated in the “Pg_vs_B0” group but was down-regulated in the transcriptome analysis. The different expression patterns presented in the qRT-PCR result and RNA-seq data were also described for some candidate genes in other studies (Li et al., 2018). Similarly, the expression level of *SgPER12* (TRINITY_DN569_c0_g1) was also different from that of RNA-Seq in the “Bg_vs_Pg” group (**Figure 7**). In a word, the correlation coefficient R^2^ values between “fold change in the qRT-PCR” and “fold change in the transcriptome” presented in the “Bg_vs_B0” group, the “Pg_vs_B0” group, and the “Bg_vs_Pg” group were 0.7807, 0.5112, and 0.8012, respectively. The results indicate that the expression profiles of DEGs obtained from RNA-seq data are reliable and efficient (**Supplementary Figure S2**).

## Discussion

In the present study, exogenous GA_3_ treatment significantly enhanced the seed vigor of *S. glauca* black seeds and then promoted seed germination rate (**Figure 1**). Transcriptome analysis showed the change in RNA levels in seed samples treated with or without GA_3_ during two critical germination stages. These RNAs are involved in plant hormone signal transduction pathways, diterpenoid biosynthesis, phenylpropanoid biosynthesis, flavonoid biosynthesis, starch and sucrose metabolism, and galactose metabolism pathways.

### Metabolism and regulation of plant hormones during GA_3_-induced dormancy release

Plant hormones play key roles during seed germination, and almost all plant hormones, such as ABA (abscisic acid), GA (gibberellic acid), CK (cytokinin), AUX (auxin), ETH (ethylene), JA (jasmonic acid), BR (brassinosteroid), and SA (salicylic acid), are involved in regulating seed germination (Yu et al., 2021). In our study, transcriptomic data revealed that some genes involved in the ABA, GA, AUX, ETH, BR, JA, and SA metabolic processes and signal transduction pathways were differentially expressed during GA_3_-induced dormancy release (**Figure 4**), implying that exogenous GA_3_ improved the germination rate of *S. glauca* black seeds by mediating the metabolic process or signal transduction of ABA, GA, AUX, ETH, BR, JA, and SA. Similar transcriptome changes were also observed in seed dormancy release induced by warm stratification in *Paris polyphylla* seeds (Liao et al., 2019) and *Amomum tsaoko* seeds (Pan et al., 2023).

It is well known that a number of genes involved in the ABA and GA signal transduction pathways affect seed dormancy (Shu et al., 2016; Yang et al., 2019). In our study, eight DEGs encoding ABA receptors (PYLs), protein phosphatases 2C (PP2Cs), and serine/threonine-protein kinases (SnRK) were enriched in the ABA signal transduction pathway (**Figure 4A**). Among them, four *SgPYL* genes exhibited differential expression patterns during GA_3_-induced dormancy release, which was consistent with the regulatory model of *PYLs* during seed germination of *Brassica juncea* (Wei et al., 2023) and *Chenopodium quinoa* (Hao et al., 2022). In addition, protein phosphatase type 2C (PP2C) is also identified as a major component of the ABA signal pathway (Rodríguez et al., 1998; Koornneef et al., 2002). In our study, three *SgPP2C* genes exhibited a decreasing trend during GA_3_-induced dormancy release, and the highest expression was presented in dry seeds (**Figure 4A**), which was consistent with the expression patterns of *FsPP2C1* (Lorenzo et al., 2001). The expression of *FsPP2C1* was negatively correlated with the germination of *Fagus sylvatica* and declined through breaking dormancy treatment (Lorenzo et al., 2001). Recent studies showed that auxin could interact with the ABA signal pathway to affect seed dormancy release (Née et al., 2017). In our study, 5 *SgSAUR* genes possessed differential expression patterns during GA_3_-induced dormancy release (**Figure 4A**), which was consistent with expression profiles of *SAUR* in Callery pear seed dormancy release induced by cold stratification (Zhang et al., 2022). In addition, our transcriptomic data revealed that five DEGs annotated as ETH, BR, JA, and SA signal transduction genes, such as *SgERS1*, *SgBR11*, *SgXTH23*, *SgTIF6B*, and *SgTGA4*, exhibited an increasing trend during GA_3_-induced dormancy release (**Figure 4A**), indicating that these genes might positively regulate dormancy release of *S. glauca* black seeds. Such *BRI1* played an important role in seed dormancy release induced by cold stratification, and overexpressing *BRI1* seeds displayed more rapid germination than wild-type (Kim et al., 2019).

Plant hormones also play an important role in seed dormancy release. For example, it was reported that GA synthesis and ABA degradation were enhanced during the dormancy release of *Arabidopsis* seeds (Footitt et al., 2011). In our study, two ABA catabolic genes, *SgCYP707A2* and *SgCYP707A4*, exhibited the highest expression levels in dry seed samples. A similar result was obtained by Sano and Marion-Poll (2021), who found that the *CYP707A2* gene had highly accumulated at the later stage of seed maturation and might play an important role in the reduction of ABA content in both the embryo and endosperm (Okamoto et al., 2006). In general, the content of ABA and the sensitivity of the embryo to ABA would decrease during the late maturation and desiccation stages (Schmitz et al., 2002). Surprisingly, the expression levels of *SgCYP707A2* and *SgCYP707A4* were significantly down-regulated in the Bg group compared with the B0 group and the Pg group (**Figure 4B**), which differed from the higher expression of *PaCYP707A1* in *Phelipanche aegyptiaca* seeds treated with FL+GA_3_ (Bao et al., 2017). Similarly, *Gh_A08G1344*, a cotton homolog of *CYP707A1*, was down-regulated during germination of XLZ38, a cold-susceptible variety (Shen et al., 2020). Morris et al. (1989) found that there was no significant association between dormancy level and seed ABA content. In addition, GA biosynthesis genes, including *SgGA3ox1* and *SgKAO1*, were significantly up-regulated during GA_3_-induced dormancy release in our study, while the GA catabolic gene, *SgGA2ox8*, was significantly down-regulated (**Figure 4C**), which was consistent with seed dormancy release induced by cold stratification in *Notopterygium incisum* (Li et al., 2018). These findings imply that exogenous GA_3_ may enhance seed endogenous GA levels by enhancing expression of GA biosynthesis genes *SgGA3ox1* and *SgKAO1* and suppressing the catabolic gene *SgGA2ox8*, further promoting the germination rate of *S. glauca* black seeds (**Figure 1B**). Yamauchi et al. (2004) also demonstrated that cold stratification-breaking seed dormancy was associated with an increase in GA content caused by the enhancement of GA20 and GA3 oxidase. Taken together, these results imply that the most important thing is not ABA degradation during GA_3_-induced dormancy release but GA synthesis, which then enhances the GA/ABA ratio and promotes seed germination. A similar result was described by Ge et al. (2023), who found that exogenous gibberellic acid promoted the germination of *Panax notoginseng* seeds by enhancing endogenous GA content and altering the GA/ABA ratio. At present, more and more studies indicate that the GA/ABA ratio has decisive and critical effects on the process of seed germination (Seo et al., 2006; Meng et al., 2016).

### Role of phenylpropanoid-related metabolism pathways during GA_3_-induced dormancy release

A previous study showed that the germination of seeds was closely connected with phenylpropanoid-related pathways (Yu et al., 2020). In our study, KEGG pathway analysis indicated that 13 DEGs were significantly enriched in the phenylpropanoid biosynthesis pathway and 6 DEGs in the flavonoid biosynthesis pathway (**Figure 5**), implying that phenylpropanoid biosynthesis and flavonoid biosynthesis are the key pathways during GA_3_-induced dormancy release. A similar result was also described by Chen et al. (2023), who found that the phenylpropanoid biosynthesis and flavonoid biosynthesis pathways had important roles in the seed germination of Chinese fir [*Cunninghamia lanceolat* (Lamb.) Hook]. In addition, most DEGs involved in the phenylpropanoid biosynthesis pathway were up-regulated during GA_3_-induced dormancy release, such as *SgANMT*, *SgHCBT2*, and *SgPER3*, etc. (**Figure 5A**). Tong et al. (2022) found that Bruceine D inhibited the germination of *Bidens pilosa* seeds by suppressing the activity of key enzymes involved in the phenylpropanoid biosynthesis pathway. Interestingly, almost half of the DEGs involved in the phenylpropanoid biosynthesis pathway were found to be peroxidase, which was consistent with the germination response of Indian mustard exposed to drought stress (Wei et al., 2023). Interestingly, except for peroxidase 3 (*SgPER3*), almost all DEGs encoding peroxidase were obtained in the genome of *S. glauca* (Yi et al., 2022). Singh et al. (2015) showed that seed germination and early axis growth depended much on the activity of peroxidase. In addition, most DEGs related to the flavonoid biosynthesis pathway were also up-regulated, such as *SgCFI3*, *SgCAMT*, *SgFLS*, etc. (**Figure 5B**). Similar to our results, the flavonoid biosynthesis pathway was significantly promoted in the dormancy release of *Polygonatum cyrtonema* seeds (Liu et al., 2021) and seed germination induced by cold stratification in *Notopterygium incisum* (Li et al., 2018). As far as we know, flavonoid and phenylpropanoid compounds play an important role in the elimination of reactive oxygen species (ROS) (Agati et al., 2009; Vogt, 2010; Fraser and Chapple, 2011).

ROS act as an important cell signal messenger, contribute to seed dormancy release, and favor subsequent seed germination. In our study, the H_2_O_2_ content of seeds treated with GA_3_ was significantly higher than that of seeds treated without GA_3_ in the initial stage of seed germination, while the opposite trend was present in the radicle emergence stage (**Figure 5C**). Similar results were reported by Jurdak et al. (2022), who found that GA_3_ alleviated *Arabidopsis* seed dormancy by inducing an overproduction of H_2_O_2_. In addition, we found that H_2_O_2_ content reached a maximum value in the initial stage of seed germination and decreased sharply with radicle emergence (**Figure 5C**). One possible explanation was that H_2_O_2_ might be converted to OH^–^ by peroxidase activity in the hydroxylic cycle, which is directly used to cell wall loosening for cell extension growth (Singh et al., 2015). Consistent with the results, the expression levels of many *SgPER* genes encoding peroxidase significantly increased during GA_3_-induced dormancy release. In contrast to H_2_O_2_ content, the O_2_^–^ content of seeds treated with GA_3_ is higher than that of seeds treated without GA_3_ in the radicle emergence stage (**Figure 5D**). A similar result was reported by Kranner et al. (2010), who found that O_2_^–^ content significantly increased with radicle elongation at the final stage of *Pisum sativum* germination. One possible explanation was that O_2_^–^ might play an important role in cell division, growth, and differentiation (Gapper and Dolan, 2006). Additionally, Kranner et al. (2010) indicated that O_2_^–^ production accompanying radicle emergence could play an important role in pathogen defense and contribute to seedling successful establishment.

### Role of carbohydrate-related metabolism pathways during GA_3_-induced dormancy release

It is well known that seed germination and growth mainly depend on carbohydrates within the seeds, which not only provide essential energy for germination but also act as osmolytes to maintain cell homeostasis (Rosa et al., 2009; Thalmann and Santelia, 2017). Krawiarz and Szczotka (2005) also showed that the seed energy metabolism was closely related to the dormancy release. In our study, KEGG pathway analysis revealed that many genes involved in starch and sucrose metabolism and galactose metabolism pathways were enriched during GA_3_-induced dormancy release (**Figure 3B**). Similar results were obtained by Zhuang et al. (2015), who found that the significant abundance changes of metabolites involved in galactose, glyoxylate, dicarboxylate, starch, and sucrose metabolism were presented in the exogenous GA_4_-induced dormancy release of flower buds in *Japanese apricot*. Similarly, Li et al. (2021) showed that GA_3_ treatment significantly changed the starch and sucrose metabolism pathways in *Leymus chinensis* seeds, which might play a key role in providing energy supply for the germination. In our study, all DEGs involved in starch and sucrose metabolism (**Figure 6A**) and galactose metabolism (**Figure 6B**) pathways were significantly up-regulated, such as *SgBAM*, *SgHXK2*, *SgSUS*, etc. Similar results were obtained by Zhang et al. (2022), who found that most DEGs involved in starch and sucrose metabolism, such as *HXK*, *SUS*, and invertase (*INV*), were significantly up-regulated during the seed dormancy release induced by cold stratification in *Pyrus calleryana* seeds.

Starch is a common polysaccharide stored in seeds that can be hydrolyzed to sugar by amylase and provide energy for seed germination. In our study, the starch content of the seeds treated with or without GA_3_ significantly decreased with the germination process, and the starch content of the seeds treated with GA_3_ was significantly lower than that of the seeds treated without GA_3_ (**Figure 6C**) in the radicle emergence stage. Similar results were obtained by Li et al. (2022), who found that GA_3_ treatment accelerated the hydrolysis of starch in *Phyllostachys edulis* seeds. Consistent with the present results, the expression levels of two key starch-hydrolases, beta-amylase (*SgBAM*) and alpha-glucosidase (*SgAGLU*), were significantly up-regulated in the Bg group (**Figure 6A**). Nandi et al. (1995) found that beta-amylase activity might play an important role in starch activation, depolymerization, and release and act as an effective index of the seed germination rate. Interestingly, we have not identified the change in alpha-amylase in our transcriptomic data, except for beta-amylase (**Figure 6A**). A similar result was obtained by Dirk et al. (1999), who found that beta-amylase significantly increased with starch hydrolysis in fenugreek seeds, but only a small amount of alpha-amylase was detected. In addition, the soluble sugar content of the seeds treated with GA_3_ was significantly lower than that of the seeds treated without GA_3_ in the initial stage of seed germination and radicle emergence (**Figure 6D**). Similar results were obtained by Wang et al. (2018), who found that exogenous GA_3_ application promoted rice seed germination by enhancing sugar consumption under low temperature conditions. Aragão et al. (2015) also showed that soluble sugar (sucrose) presented the highest content in mature seeds of *Cedrela fissilis* but decreased significantly during germination. Consistent with the change in soluble sugar content, the expression level of the *SgHXK2* gene, encoding the hexokinase, was significantly induced during GA_3_-induced dormancy release (**Figure 6**). Zhao et al. (2019) showed that the relative expression level of *PbHXK1* was negatively correlated with sugar content during pear fruit development but significantly positively correlated with hexokinase activity. Furthermore, the overexpression of the *Arabidopsis* hexokinase 1 gene (*AtHXK1*) in tomato plants significantly decreased the starch and sugar content of transgenic fruit (Menu et al., 2003).

## Conclusion

**Figure 8:**
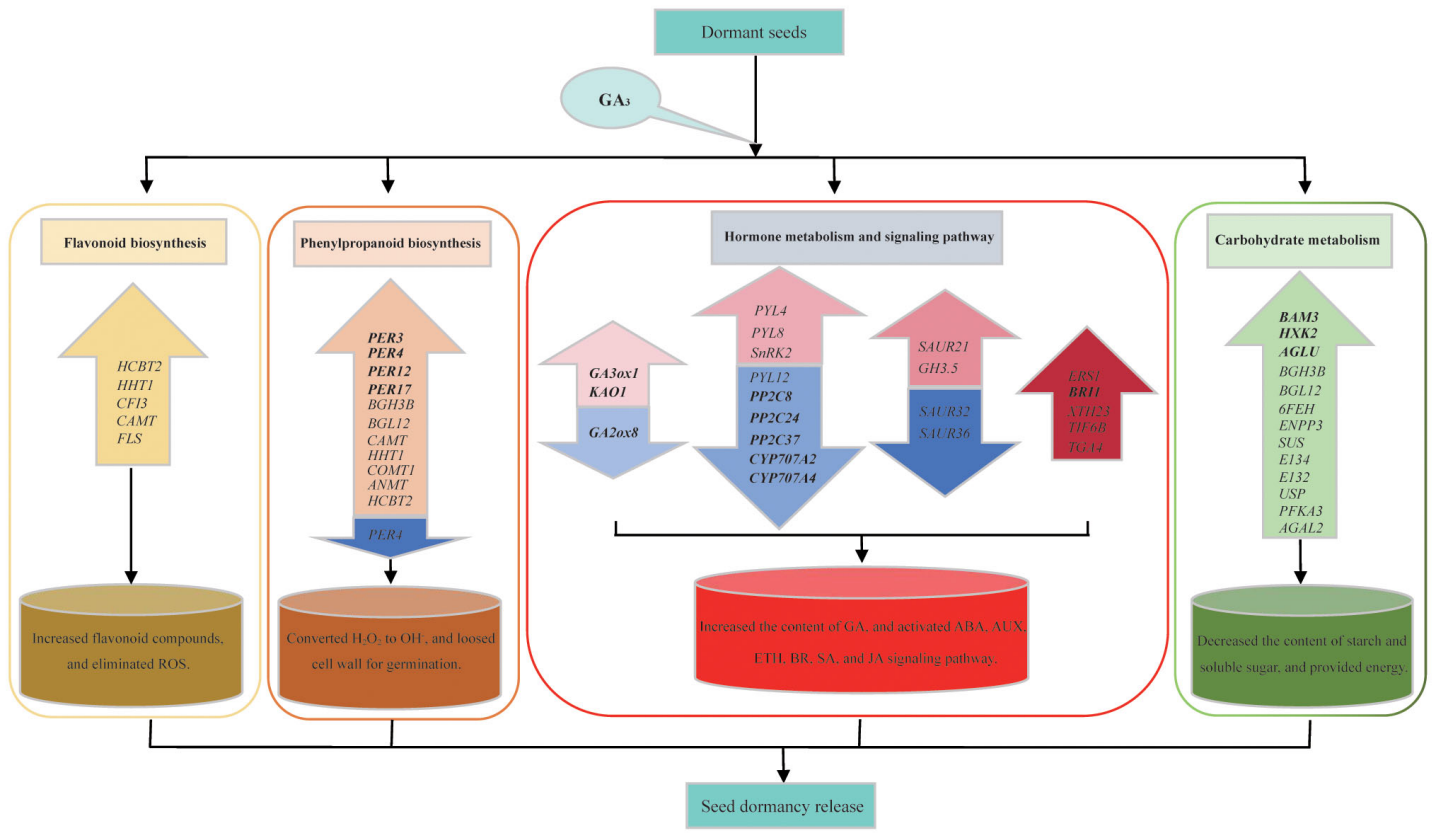
Regulation model of exogenous GA_3_-induced dormancy release on *S. glauca* black seeds. The colored up-arrow indicates the up-regulation of genes involved in the related pathway, and the colored down-arrow indicates the down-regulation of genes involved in the related pathway.

There are significant alterations in seed vigor, H_2_O_2_ and O_2_^–^ contents, as well as starch and soluble sugar contents, during exogenous GA_3_-induced seed germination, which reflect the physiological changes in the dormancy release process in *S. glauca* black seeds. Comparative transcriptome analysis indicates that a total of 1136 co-expressed DEGs are identified in three comparison groups. Among them, plant hormone signal transduction pathways are one of the main pathways enriched in the KEGG pathway, and genes related to auxin (*SgSAURs*) and ABA (*SgPYLs* and *SgPP2Cs*) signal transduction and GA metabolism (*SgGA3ox1, SgKAO*, and *SgGA2ox8*) are identified as the main targets for GA_3_-induced dormancy release. The genes involved in the phenylpropanoid and flavonoid biosynthesis pathways are activated, contributing to maintaining ROS homeostasis in GA_3_-induced dormancy release, which is consistent with the changes in H_2_O_2_ and O_2_^–^ content. The genes related to the starch and sucrose pathways as well as the galactose pathway are also activated and provide essential energy sources for GA_3_-induced seed germination, which is consistent with the changes in starch and soluble sugar content. In a word, a regulatory model related to GA_3_-induced dormancy release in *S. glauca* black seeds was proposed according to the transcriptome analysis and physiological indicator analysis (**Figure 8**). Exogenous GA_3_ application can increase the content of endogenous GAs by inducing the expression of GA metabolism enzyme genes, altering the ratio of GA/ABA, promoting the production of ROS, which in turn activate non-enzyme antioxidant systems, maintaining ROS homeostasis, and enhancing the activities of related starch-hydrolyse enzymes to hydrolyze stored starch, then providing an energy source. These results reveal the synergistic effect of genes related to the plant hormone signal transduction pathway, the phenylpropanoid and flavonoid biosynthesis pathways, and the carbohydrate metabolism processes (starch and sucrose pathways and galactose pathways), which will contribute to promoting seed dormancy release in *S. glauca* black seeds. The findings obtained in this study will strengthen our understanding of the molecular mechanisms of GA_3_-induced dormancy release in *S. glauca* black seeds and further enrich our knowledge of seed dormancy and germination.

## Data availability statement

The datasets presented in this study can be found in online repositories. The names of the repository/repositories and accession number(s) can be found in the article/**Supplementary Material**.

## Author contributions

HW: Conceptualization, Funding acquisition, Methodology, Project administration, Supervision, Writing – original draft. TX: Data curation, Investigation, Software, Writing – original draft. YL: Data curation, Investigation, Writing – original draft. RG: Data curation, Resources, Writing – original draft. XT: Data curation, Writing – original draft. JS: Software, Writing – original draft. CL: Data curation, Writing – original draft. QL: Conceptualization, Funding acquisition, Project administration, Supervision, Writing – review & editing.
