# Supplementary material for: Comparative transcriptome analysis reveals the potential mechanism of GA3-induced dormancy release in Suaeda glauca black seeds
Source: Front Plant Sci. 2024 Jun 11;15:1354141. doi: 10.3389/fpls.2024.1354141 (PMC11197467; doi:10.3389/fpls.2024.1354141)
Supplement: Supplementary file 2 [file DataSheet_1.docx]

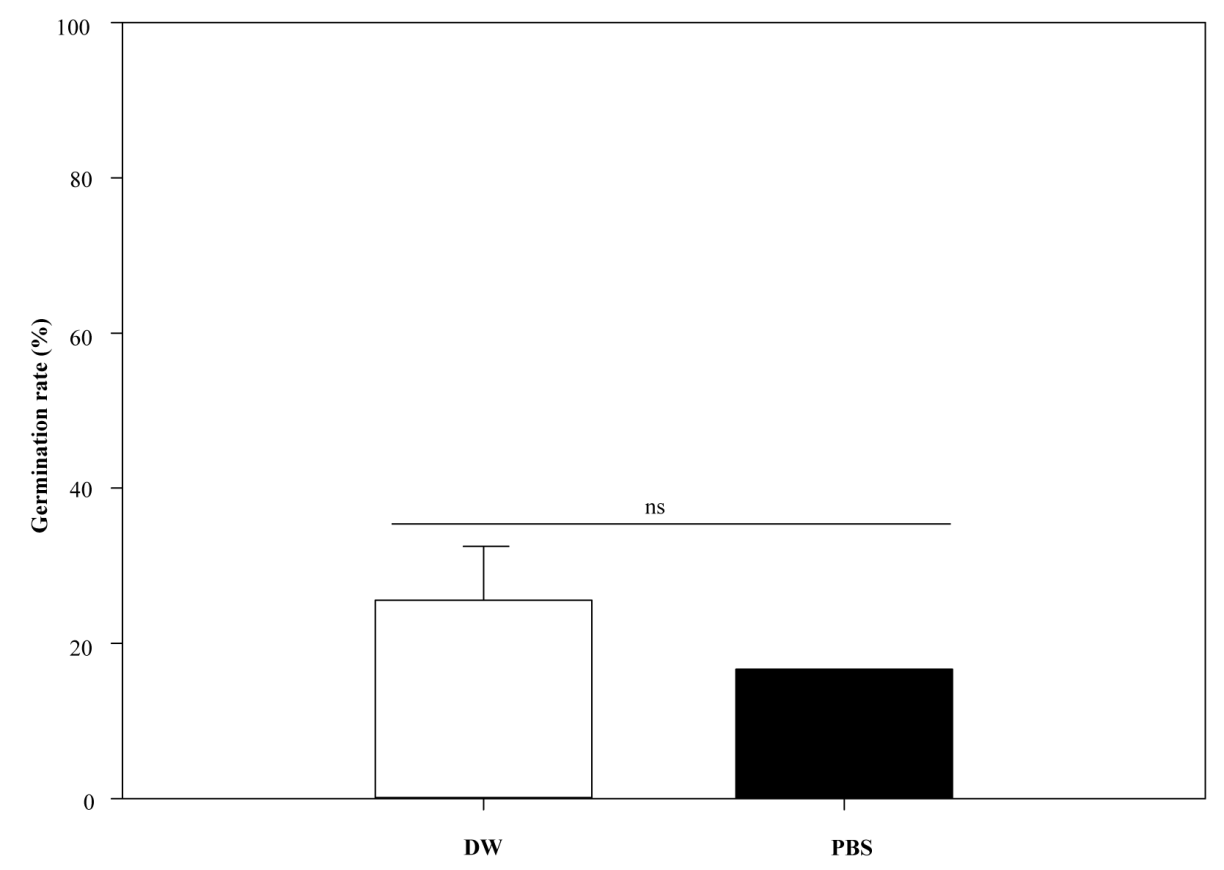


Figure S1. Effects of phosphate buffered solution (PBS, 0.01 mol/L) and distilled water (DW) on seed germination. “ns” represents no difference according to t-test. Vertical bars indicate means ± SD (n=3).


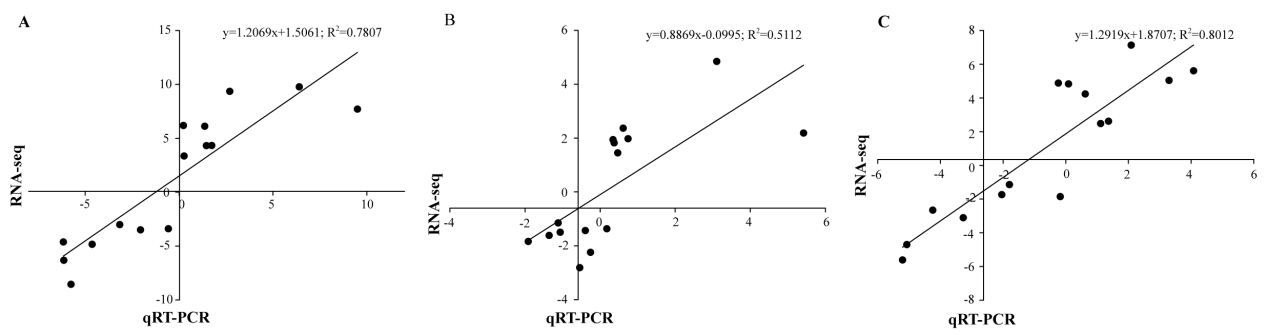


Figure S2. Correlation plot of the qRT-PCR results and RNA-seq results in Bg_vs_B0 group (A), Pg_vs_B0 group (B), and Bg_vs_Pg group (C). Results were obtained by using log2 fold change measurements.
